# A Word for Nitrous Oxide

**Published:** 1872-04

**Authors:** 


### A WORD FOR NITROUS OXIDE.

BY A. W. S., BOSTON.

It has been well remarked that God never suspends the laws of nature to accommodate our ignorance. Neither piety nor learning can prevent the physiological action of a poison administered to the system through ignorance.

The want of discrimination and an exercise of common sense gives birth to many a blunder which is sired upon a pure and harmless agent.

It is human nature to shirk the responsibility of mistakes. We have noticed this particularly in professional practice. Physicians never err, but the chemist and apothecary do this, how often? The Dentist, especially, with a diploma, is wholly right in his operations—bad results are due to the idiosincrasies of the patient, the peculiar formation of the mouth, defective mechanical or chemical appliances, etc. That bad results often follow the skillful use of bad agents no one will deny. Yet experience and observation prove that such results oftener follow from ignorance or want of skill in the use of agents really good. *Per Se.*

This is especially true in the use of the present popular anæsthetic—Nitrous Oxide. Experiences with this vary more than with any other agent employed for assuaging the pains of surgical operations. Dr. A., of undoubted veracity, has extracted wholly with the gas for five years, having given it over 12,000 times without a single really bad result—would not think of doing without it.

Dr. B. has the most approved apparatus yet devised for preparing gas (two small filthy washers, a cask, and an Irish boy). Besides it has been examined by Professor Fee, the State Assayer, the gas tested (smelt of) and in a well paid for certificate pronounced all right. And yet patients are nauseated, blackened in the face, thrown into convulsions, and started with a consumptive cough.

Dr. C. who has a good apparatus for generating and inhaling—has judgment and skill—looks after it himself, understands its operations, and complies strictly with the conditions for its success, writes: "I have had no trouble in preparing or administering the gas, think it decidedly more safe and satisfactory than ether. My patients all like it—won't take any other anæsthetic."

Prof. D., the author of a school-book of chemistry, thinks Nitrous Oxide, should be given only in the presence of an M. D. Has made and inhaled it more than once by his process (a pint washer and a bladder) and never without experiencing a degree of vertigo, followed by nausea.

Thus, doctors disagree, and opinions vary. Who shall decide? If I may be allowed to refer to my own experience it is, that after having inhaled this gas some two hundred times to anæsthesia, I have never yet experienced nausea or any of the bad effects too often ascribed to the pure gas. and while I have often administered it to others it has never been with the experience occasionally reported in journals. I have, however, been discriminating as to the quality of gas, manner of inhaling, and physiological condition of the patient. My inference, therefore is, that when properly prepared, properly and skillfully administered, Nitrous Oxide may be pronounced as safe and as economical as any anæsthetic yet discovered.

Practical intelligence in the nature and use of anæsthetics is a want with the Dental Profession, in many cases at least.

The lack of chemical knowledge has led many to follow jack o-lanterns in science, and, when out of the bog again, they have been led to make condemnations.

For example, many dentists were caught sometime since with the plan of using the gas condensed in metallic cylinders, because it had the sanction of a professor who knows much more of gaseous generalties than of gaseous specialties.

Economy and safety in the use of anæsthetics, are in the direct line of skill and intelligence. Let reasonable care be used in preparing Nitrous Oxide, and judgment be shown in administering it, and it is questionable if any anæsthetic can be found to supersede it in the matter of both safety and economy, especially at the present low price of material.

